# Supplementary material for: Molecular robotic agents that survey molecular landscapes for information retrieval
Source: Nat Commun. 2024 Apr 17;15:3293. doi: 10.1038/s41467-024-46978-2 (PMC11024175; doi:10.1038/s41467-024-46978-2)
Supplement: Supplementary file 1 — Supplementary Information [file 41467_2024_46978_MOESM1_ESM.pdf]

Supplementary Information for

Molecular robotic agents that survey molecular landscapes for information retrieval

Sungwook Woo*, Sinem K. Saka, Feng Xuan, Peng Yin*

Correspondence to: swoo@postech.edu, py@hms.harvard.edu

Table of Contents

Supplementary Figure 1. Sequence diagram of the rectangle origami used in this study. 2

Supplementary Figure 2. Probe position maps for different tests presented in the main text........ 3

Supplementary Figure 3. AFM images of individual origami rectangles used to obtain average images........ 4

Supplementary Figure 4. Images of full gel lanes with band length annotations. 5

Supplementary Figure 5. 'Scalability' in terms of the number of steps taken by crawlers 6

Supplementary Figure 6. Probe architecture variations. 7

Supplementary Figure 7. Gel-based 'calibration' for estimation of pre-amplification record amount 8

Supplementary References 9

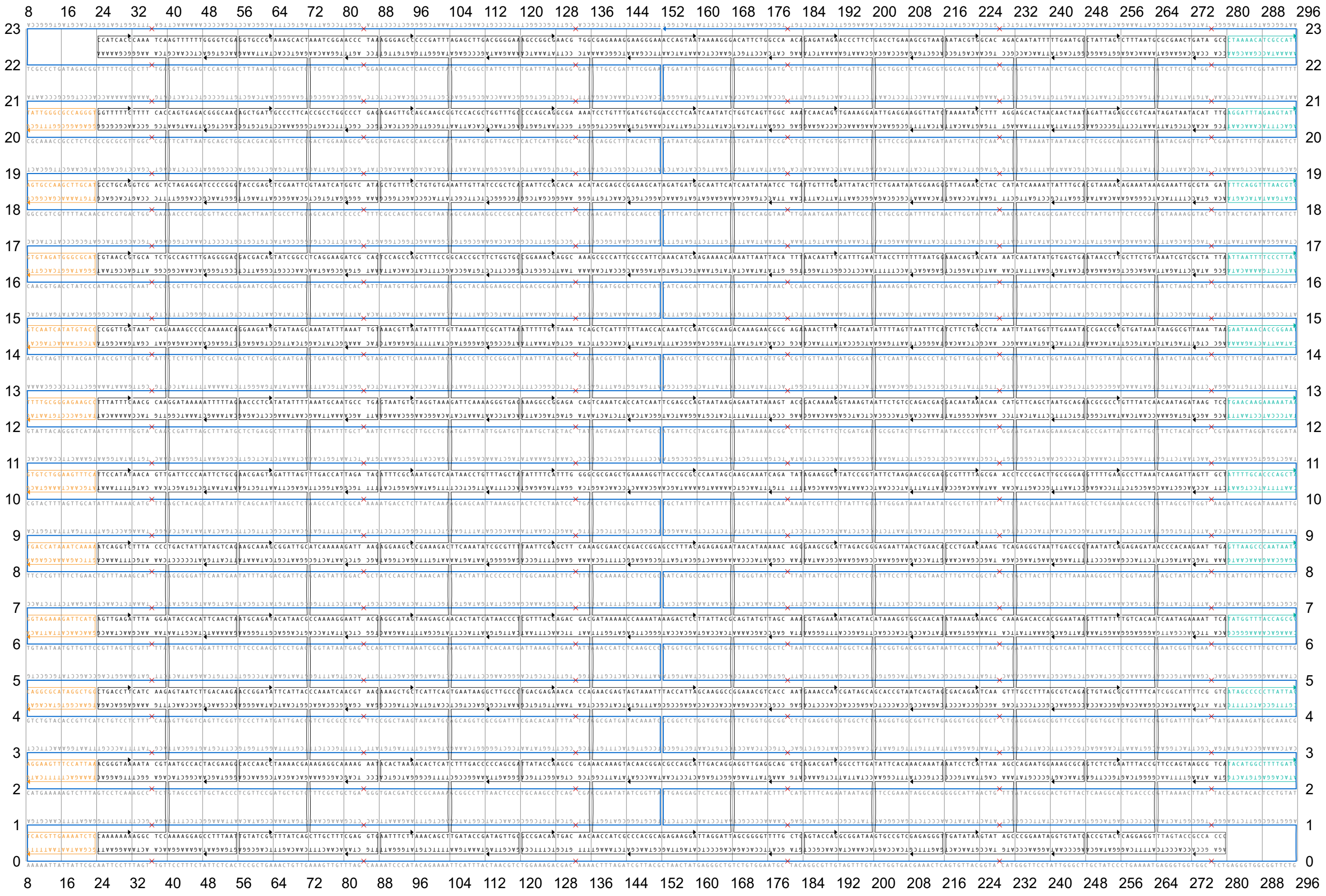

Supplementary Figure 1. Sequence diagram of the rectangle origami used in this study. The blue lines show the scaffold routing path and the black strands represent staples strands. The orange and cyan strands on the left and right, respectively, are edge staples that allow the origami to stack and form a long chain. The edge staples were typically omitted for most experiments, but were included in AFM tests to create long chains, in order (1) to allow tighter binding to the mica surface and (2) to facilitate finding the same set of origami before and after recording from large-area scans. 11 edge staples from each side out of 12 were used and placed such that they create an asymmetric offset in chains, which helped identify the landing side (top vs bottom) of the origami in a given chain. The numbers at the top and bottom of the diagram indicate the base numbers and those at the left and right edges denote the helix numbers. Red “×” marks represent the deletion sites introduced for twist correction.

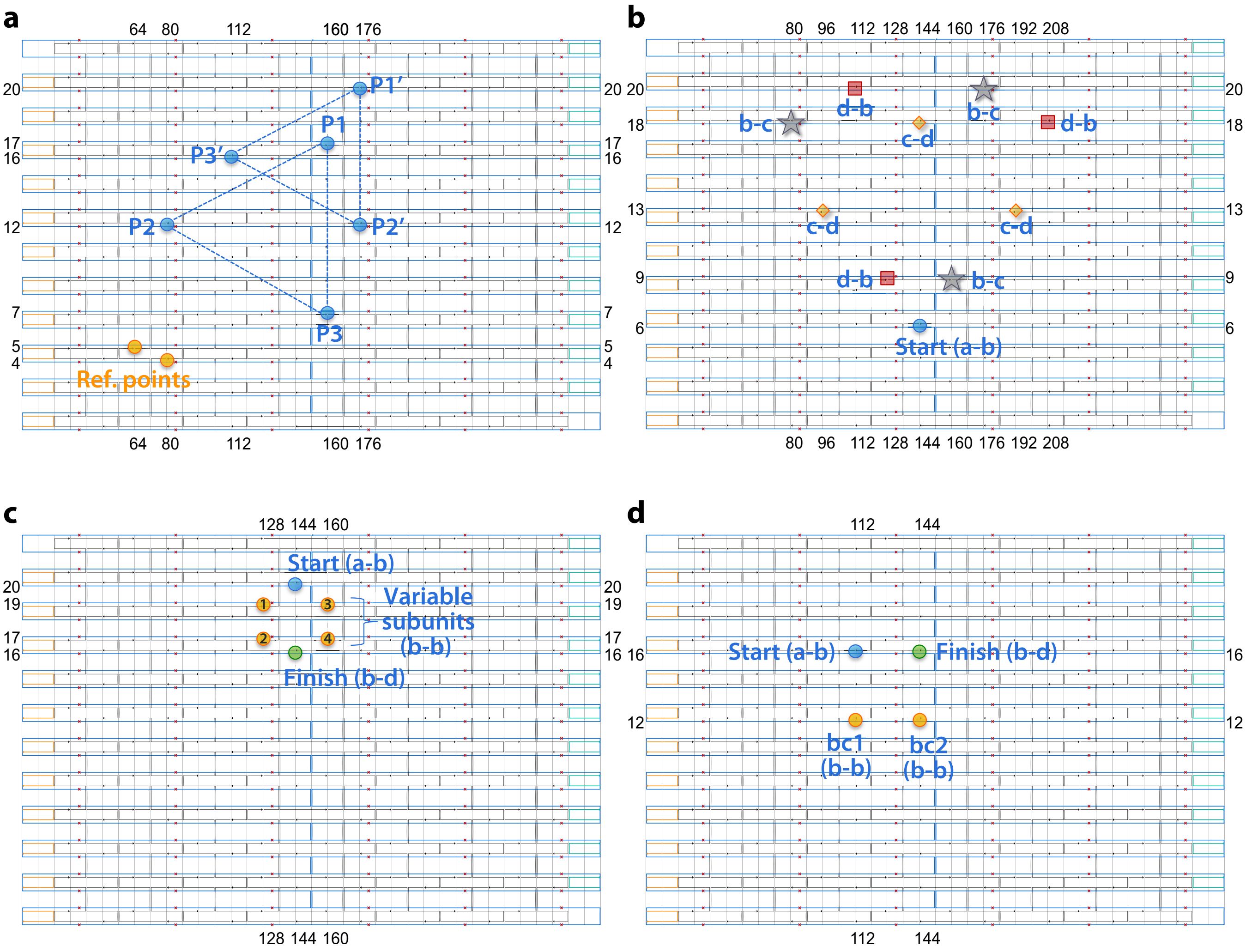

Supplementary Figure 2. Probe position maps for different tests presented in the main text. **a**, Three-point tests. The blue circles represent the positions of sets of three probes (two versions, simply for historical reasons; the triangles marked with P1-P2-P3 and with P1'-P2'-P3' were used for tests shown in Figs. 2a-c and in Figs. 2d,e, respectively.) and the orange circles denote the reference marks placed on origami. The distance between the reference points is ~6 nm. **b**, Ten-probe scalability tests. Probes were placed such that a crawler initiates a reaction at the start (a-b) site, then proceed to a nearby b-c probe, then to c-d, and to d-b, then to the next b-c and continue in that order. **c**, Artificial complex size measurement tests. For complex sizes 3, 4, and 5 presented in Fig. 3f, in addition to the start and finish sites, variable subunits at position 1, at positions 1 and 2, and at positions 1, 2, and 4 were used, respectively. **d**, Four-probe path 'deconvolution' tests. The numbers at the top and bottom of each diagram indicate the base numbers and those at the left and right edges denote the helix numbers, as in Supplementary Figure 1.

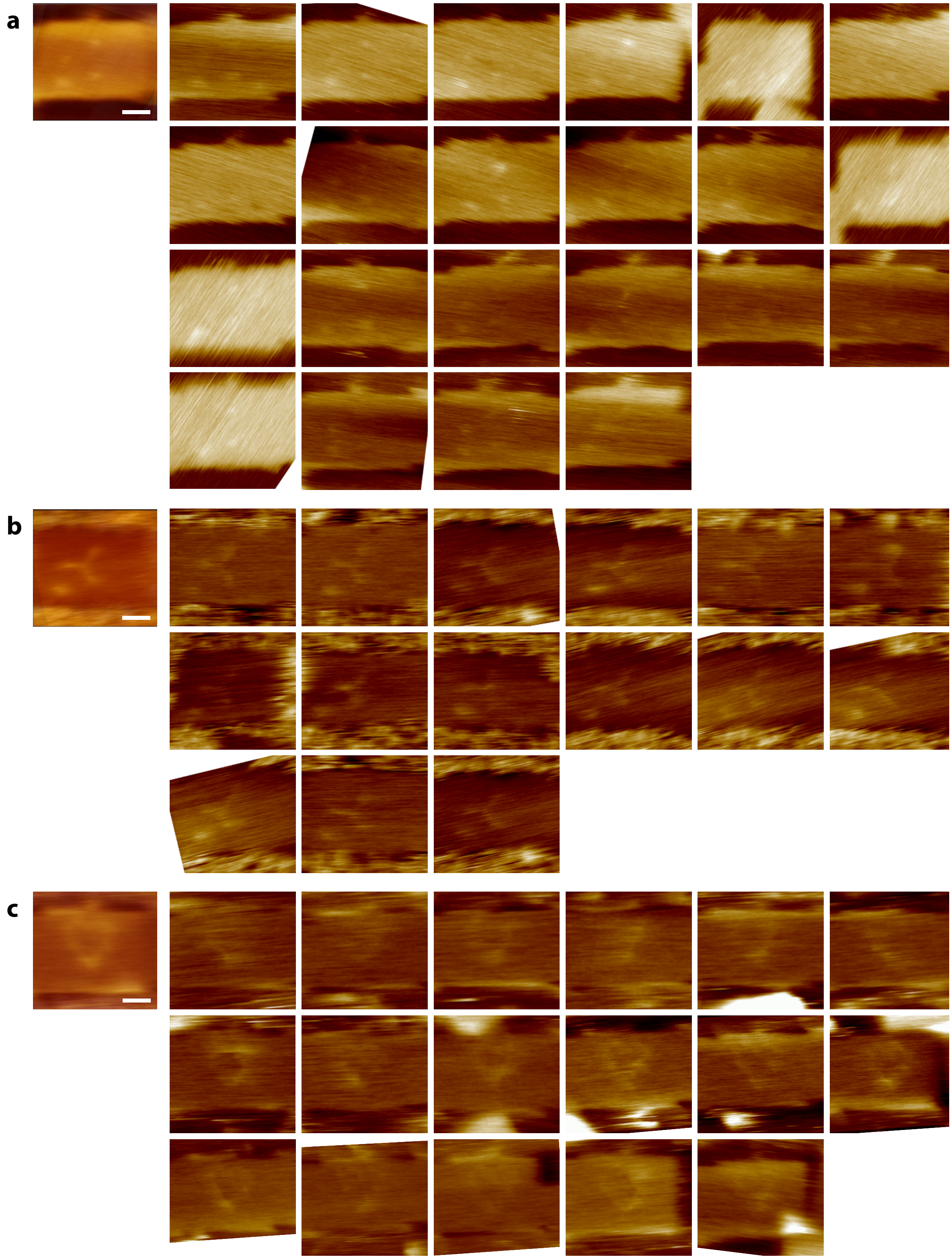

Supplementary Figure 3. AFM images of individual origami rectangles (all different molecules) used to obtain average images.
a-b, Before (**a**) and after (**b**) recording of the three-point tests (Fig. 2b, (ii) and (iv)). **c**, After recording of the ten-probe scalability tests (Fig. 2f(iii)). Leftmost are average images. Scale bars, 25 nm.

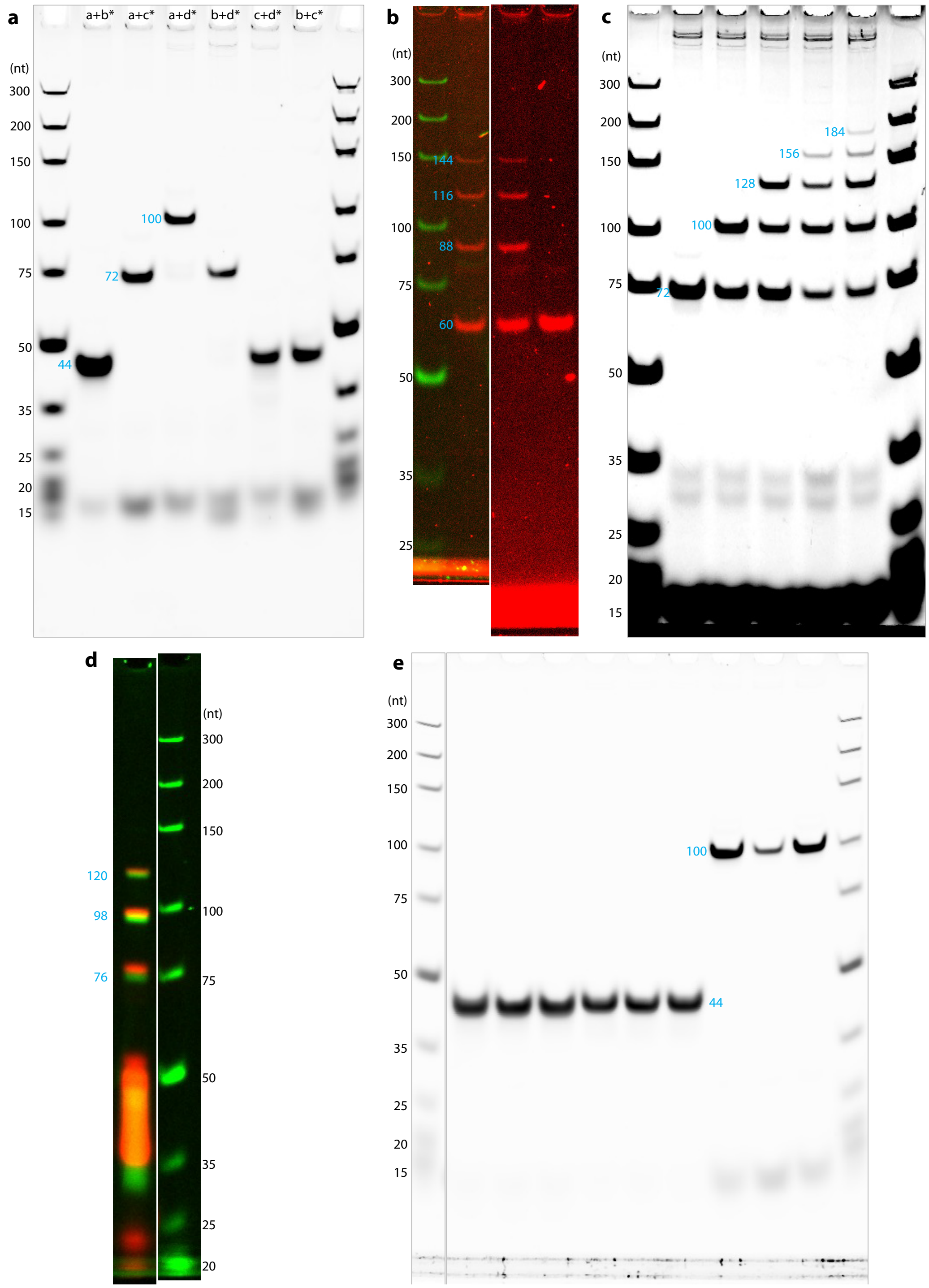

Supplementary Figure 4. Images of full gel lanes with band length annotations.
a, Full gel lanes for Fig. 2c in the main text, including additional lanes for PCR products for subsegments of the record. PCR primers used for each lane were marked at the top of the gel image. Lengths for key bands in nt (nucleotides) are indicated in blue text next to the respective bands. **b**, Full gel lanes for Fig. 3c along with another gel for the same sample that contains a ladder. **c**, Full gel lanes for Fig. 3f. **d**, Full gel lanes for Fig. 3h. The red smear between ~35 and ~50 is due to a degenerate-base barcode added in the first probe. **e**, Full gel lanes for Fig. 4c. For (**b**) and (**d**), red signal is from Cy5 and green from SYBR Gold. All other gel images are from SYBR Gold staining. Source data are provided as a Source Data file.

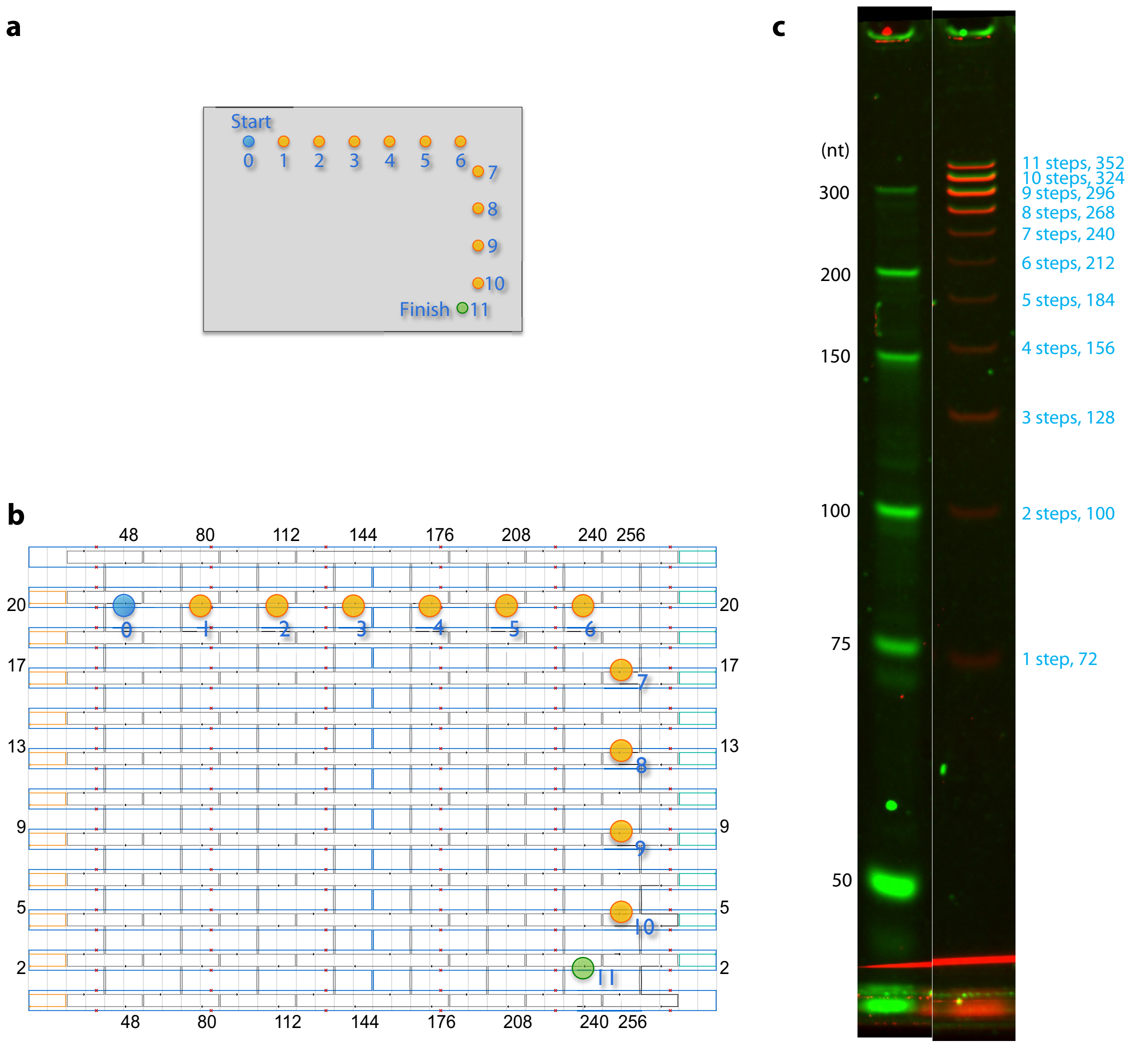

Supplementary Figure 5. 'Scalability' in terms of the number of steps taken by crawlers. **a-b**, Schematic diagram (**a**) and probe position map (**b**) of a long-track origami with 12 track sites, with a 'start' site at position 0, a 'finish' site at position 11, and probes with 'repeating' primers (as described in Fig. 3 in the main text) in between. The numbers in black at the top and bottom of the map in (**b**) indicate the base numbers and those at the left and right edges denote the helix numbers, as in Supplementary Figure 1. **c**, Gel data showing records generated from the track, after PCR amplification. Crawlers reach the full 11 steps, where the number of track positions is primarily limited by the size of the origami testbed, and should in principle keep crawling if there are more track sites, as long as molecular resources (e.g., nucleotides) are constantly supplied. Note that the records with fewer step numbers result from occasional 'skips' allowed by the random crawling mechanism enabled by the repeating primers by design. The number of steps and the corresponding length in nt (nucleotides) for each band is indicated in blue text next to the gel. Red signal is from Cy5 and green from SYBR Gold. This test was repeated three times with the identical or similar conditions, which gave equivalent results. Source data are provided as a Source Data file.

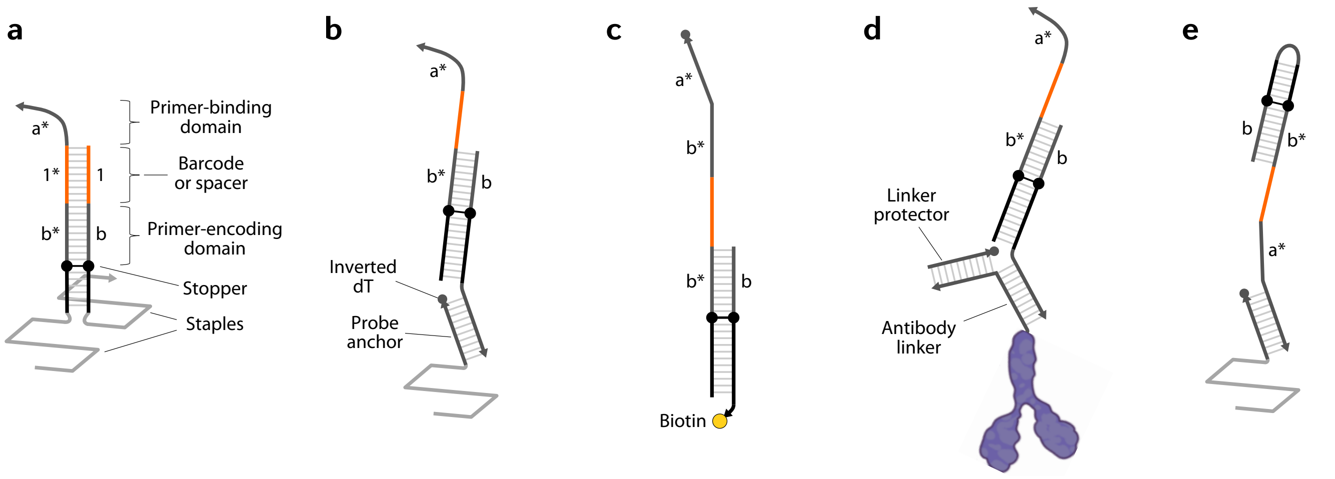

Supplementary Figure 6. Probe architecture variations.
While sharing the same basic core structure and principle, a few different variations of probe/anchor design architectures were used in this study. **a**, Probe pair incorporated as an extension from staple strands. This version was used for the three-point track test as shown in Figs. 2a-c. Stopper was implemented either by a noncanonical base pair iso-dC/iso-dG or a non-DNA spacer (/iSp9/) placed opposite to a T (see sequence list for details); earlier designs used iSp9 and later designed adopted the iso-dC/iso-dG pair for improved kinetics[1]. **b**, A modified version of probe/anchor design for improved modularity; each probe can be linked to origami via just one staple as opposed to two as in (**a**). Barcode/spacer domain is turned single-stranded as well to improve modularity (the opposite side can simply have fixed sequences) and to ease sequence design constraints (e.g., potentially introducing degenerate code). The 3' end of the staple with anchor extension is protected against elongation by polymerase by the added inverted dT (/3InvdT/). This version was used for tests presented in Figs. 2d-f and 3g-j. **c**, The 'bottom' strand of a probe duplex can be designed to have different types of linkage moieties. This version containing biotin was used for the streptavidin subunit counting tests as shown in Figs. 3a-c. **d**, The architecture described in (**b**) was used for the microtubule tests (Fig. 4) to link probe duplexes with antibodies through an 'antibody linker' strand. The antibody linker strands that were pre-attached to antibodies for general purposes in the lab had a long length (42 bp), so the remaining portion was protected by a 'linker protector' which was capped by an inverted dT at the 3' end. The antibody portion of the figure was adapted from Fig. 4a, which was prepared using BioRender.com. **e**, An 'inverted' design used for further improved modularity. A probe duplex is replaced with just a single hairpin probe, by moving the anchor-binding domain to the 3' end stretch of the primer-binding domain. This version was used for the artificial complex counting tests as shown in Fig. 3d-f.

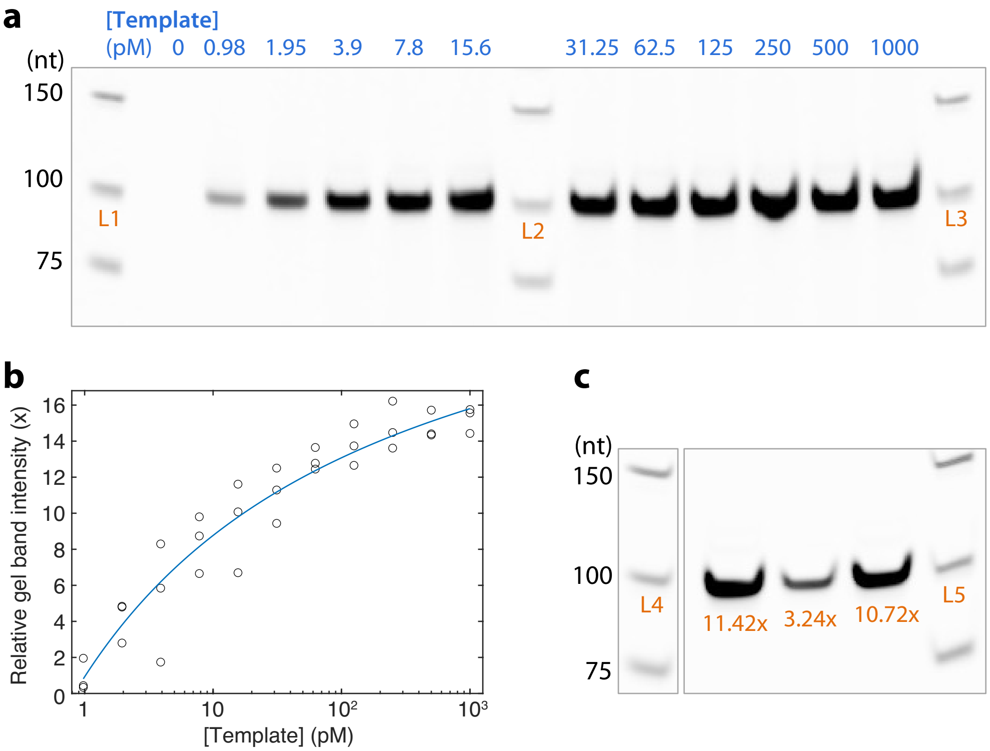

Supplementary Figure 7. Gel-based 'calibration' for estimation of pre-amplification record amount.

a, Full record product of a known concentration was serially diluted, then PCR-amplified and run in a denaturing gel. The band intensities of the products were measured, then normalized by the average of the ladder bands at the same length (L1, L2 and L3) to obtain the 'relative' gel band intensities. nt, nucleotides. **b**, Plot of thus-obtained relative gel band intensity versus the pre-amplification template concentration (for three independent tests), along with a fit based on the rat11 model in MATLAB (typical model for saturation curves; $y = (27.9177x + 2.2622) / (x + 2.4425)$, $R^2 = 0.9167$). **c**, The gel bands of the trivalent records presented in Fig. 4c were analyzed the same way to obtain their 'relative' intensities as normalized by the average of the ladder bands in that gel (L4 and L5); the calculated relative intensities are displayed with orange numbers in the figure. Using the fit from (**b**), the pre-amplification record concentrations were estimated to be ~35.75, ~1.70, and ~24.65 pM, for the three product bands, respectively. Gel images are from SYBR Gold staining. Source data are provided as a Source Data file.

Supplementary References

1. Schaus, T. E., Woo, S., Xuan, F., Chen, X. & Yin, P. A DNA nanoscope via auto-cycling proximity recording. *Nat. Commun.* **8,** 696 (2017).
