# Supplementary material for: Molecular robotic agents that survey molecular landscapes for information retrieval
Source: Nat Commun. 2024 Apr 17;15:3293. doi: 10.1038/s41467-024-46978-2 (PMC11024175; doi:10.1038/s41467-024-46978-2)
Supplement: Supplementary file 3 — Description of additional supplementary files [file 41467_2024_46978_MOESM3_ESM.pdf]

DESCRIPTION OF ADDITIONAL SUPPLEMENTARY FILES DOCUMENT

Supplementary Data 1. Sequences of plain origami staple strands

Supplementary Data 2. Sequences of origami staple strands with extensions for probes/anchors

Supplementary Data 3. Sequences of probes, anchors, and other strands
