# Supplementary material for: Molecular robotic agents that survey molecular landscapes for information retrieval
Source: Nat Commun. 2024 Apr 17;15:3293. doi: 10.1038/s41467-024-46978-2 (PMC11024175; doi:10.1038/s41467-024-46978-2)
Supplement: Supplementary file 5 — Reporting Summary [file 41467_2024_46978_MOESM5_ESM.pdf]

nature portfolio

Corresponding author(s): Sungwook Woo & Peng Yin

Last updated by author(s): Mar 13, 2024

Reporting Summary

Nature Portfolio wishes to improve the reproducibility of the work that we publish. This form provides structure for consistency and transparency in reporting. For further information on Nature Portfolio policies, see our Editorial Policies and the Editorial Policy Checklist.

Statistics

For all statistical analyses, confirm that the following items are present in the figure legend, table legend, main text, or Methods section.

n/a	Confirmed	
☐	☒	The exact sample size (*n*) for each experimental group/condition, given as a discrete number and unit of measurement
☐	☒	A statement on whether measurements were taken from distinct samples or whether the same sample was measured repeatedly
☐	☒	The statistical test(s) used AND whether they are one- or two-sided<br>*Only common tests should be described solely by name; describe more complex techniques in the Methods section.*
☒	☐	A description of all covariates tested
☒	☐	A description of any assumptions or corrections, such as tests of normality and adjustment for multiple comparisons
☐	☒	A full description of the statistical parameters including central tendency (e.g. means) or other basic estimates (e.g. regression coefficient) AND variation (e.g. standard deviation) or associated estimates of uncertainty (e.g. confidence intervals)
☐	☒	For null hypothesis testing, the test statistic (e.g. *F*, *t*, *r*) with confidence intervals, effect sizes, degrees of freedom and *P* value noted<br>*Give P values as exact values whenever suitable.*
☒	☐	For Bayesian analysis, information on the choice of priors and Markov chain Monte Carlo settings
☒	☐	For hierarchical and complex designs, identification of the appropriate level for tests and full reporting of outcomes
☐	☒	Estimates of effect sizes (e.g. Cohen's *d*, Pearson's *r*), indicating how they were calculated

Our web collection on statistics for biologists contains articles on many of the points above.

Software and code

Policy information about availability of computer code

Data collection	Gel images were obtained on Typhoon FLA 9000 (GE), FluorChem M (ProteinSimple) or Azure C600 (Azure Biosystems) with their respective software provided by the manufacturers (Typhoon software and FluorChem software version numbers unknown; Azure cSeries Capture Software, ver. 1.9.4.0517). AFM images were taken using a Multimode AFM (Veeco Metrology Group) with a Nanoscope V controller and the accompanying software (version number unknown). Quantitative PCR data were acquired using QuantStudio 1 (Thermo Fisher) and processed using the software from the manufacturer (QuantStudio Design & Analysis Software, ver. 1.5.2). Fluorescence microscopy images were obtained from Leica DMI6000B with the software provided (version number unknown).
Data analysis	Gel data and fluorescence images were analyzed using ImageJ (ver. 2.1.0) and additional post-processing (e.g., averaging) was performed using MATLAB (R2021a or R2023b). Averaging of AFM images and plotting of graphs were performed using MATLAB. High-throughput sequencing data were analyzed using a combination of shell commands, MATLAB, and Google Sheets. Statistical tests and curve fitting were performed using MATLAB.

For manuscripts utilizing custom algorithms or software that are central to the research but not yet described in published literature, software must be made available to editors and reviewers. We strongly encourage code deposition in a community repository (e.g. GitHub). See the Nature Portfolio guidelines for submitting code & software for further information.

Data

Policy information about availability of data

All manuscripts must include a data availability statement. This statement should provide the following information, where applicable:

- Accession codes, unique identifiers, or web links for publicly available datasets
- A description of any restrictions on data availability
- For clinical datasets or third party data, please ensure that the statement adheres to our policy

The high-throughput sequencing data generated in this study have been deposited in the National Center for Biotechnology Information (NCBI) Sequence Read Archive (SRA) database under accession code SRR28124321 [https://www.ncbi.nlm.nih.gov/sra/SRR28124321]. All other data are provided in the Source Data file that accompanies this paper.

Research involving human participants, their data, or biological material

Policy information about studies with human participants or human data. See also policy information about sex, gender (identity/presentation), and sexual orientation and race, ethnicity and racism.

Reporting on sex and gender	No human participants were recruited, and no human data were collected. Cell lines used were commercial products derived from African green monkey's kidney, where sex information is unknown or irrelevant.
Reporting on race, ethnicity, or other socially relevant groupings	No human participants were recruited, and hence no social classification was performed.
Population characteristics	See above.
Recruitment	No human participants were recruited.
Ethics oversight	Not relevant.

Note that full information on the approval of the study protocol must also be provided in the manuscript.

Field-specific reporting

Please select the one below that is the best fit for your research. If you are not sure, read the appropriate sections before making your selection.

☒ Life sciences ☐ Behavioural & social sciences ☐ Ecological, evolutionary & environmental sciences

For a reference copy of the document with all sections, see nature.com/documents/nr-reporting-summary-flat.pdf

Life sciences study design

All studies must disclose on these points even when the disclosure is negative.

Sample size	For experiments where comparison between groups was necessary (the microtubule tests; as in Fig. 4d), the sample size (n=8) was chosen to ensure that the differences in gel intensity between groups become statistically significant, based on t-tests. For the microscopy experiments, no statistical tests are possible because the comparison between groups is qualitative (in terms of the distribution of fluorescence, or the pattern of fluorescence intensity profile), and with the goal of checking consistency with the gel-based results, two independent experiments were performed, both of which confirmed consistency; within each repetition, 35, 32 and 33 different cells were analyzed with 387, 174 and 504 intensity profiles for groups (1), (2) and (3), respectively.
Data exclusions	No data were excluded from the analyses unless otherwise noted; NGS reads missing essential primer sequences, which amount to ~5.9% of the total reads, were excluded due to the sequence error not allowing proper sequence parsing and analysis.
Replication	For tests where no comparison between groups is necessary, no statistical method was used to predetermine repetition size, but all experiments were repeated multiple times, with all attempts yielding consistent results. Specifically, for data in Fig. 2b, tests were repeated four times; for data in Fig. 2c, tests were repeated at least five times to confirm the results and to use as a reference for other tests; for data in Fig. 2f, AFM imaging for that particular sample was performed once, but similar tests with other patterns (e.g., line; data not shown) were performed multiple times (at least five times) altogether; for data in Fig. 3c, tests were repeated eight times or more with the identical or similar conditions to confirm the results and to use as a reference for other tests; for data in Fig. 3f, tests were repeated five times with the identical or similar conditions; for data in Fig. 3h, tests were repeated at least five times with the identical or similar conditions.
Randomization	The experiments were not randomized, but where the cell population was divided into three groups for respective treatments, no pre-assignment or selection procedure was applied, and each population was taken from the same source through consecutive and consistent pipetting. Likewise, in the recording experiments, the incubation solutions were prepared from the same master mixes and applied in parallel through consecutive and consistent pipetting.
Blinding	The investigators were not blinded to allocation during experiments and outcome assessment; for most experiments in this study, blinding

Blinding	was not relevant, because the tests were run in parallel (as in gel experiments). For microscopy experiments, blinding was practically not possible, because the sample prep, data acquisition and analysis were performed by the same investigator(s).

Reporting for specific materials, systems and methods

We require information from authors about some types of materials, experimental systems and methods used in many studies. Here, indicate whether each material, system or method listed is relevant to your study. If you are not sure if a list item applies to your research, read the appropriate section before selecting a response.

Materials & experimental systems

n/a	Involved in the study
☐	☒ Antibodies
☐	☒ Eukaryotic cell lines
☒	☐ Palaeontology and archaeology
☒	☐ Animals and other organisms
☒	☐ Clinical data
☒	☐ Dual use research of concern
☒	☐ Plants

Methods

n/a	Involved in the study
☒	☐ ChIP-seq
☒	☐ Flow cytometry
☒	☐ MRI-based neuroimaging

Antibodies

Antibodies used	- Rat alpha-tubulin antibody (Invitrogen, Cat. No. MA1-80017, Clone YL1/2)<br>- Mouse beta-tubulin antibody (Developmental Studies Hybridoma Bank, Cat. No. E7, Clone E7)<br>- Rabbit EB-1 antibody (Sigma, Cat. No. E3406, polyclonal)<br>- Anti-mouse secondary antibody (Jackson ImmunoResearch Cat. No. 715-005-151, polyclonal)<br>- Anti-rabbit secondary antibody (Jackson ImmunoResearch Cat. No. 711-005-152, polyclonal)
Validation	All antibodies used are from commercial sources, with validation data including immunofluorescence and immunoblot results and relevant references (at least 20 for each as of March 2023) available from their respective web pages. Below is a list of one reference for each antibody as an example:<br>(1) rat alpha-tubulin antibody – doi: 10.1038/ncomms13874<br>(2) mouse beta-tubulin antibody – doi: 10.1101/pdb.prot105635<br>(3) rabbit EB-1 antibody – doi: 10.1038/ncomms11665<br>(4) anti-mouse secondary antibody – doi: 10.1038/s41467-022-32626-0<br>(5) anti-rabbit secondary antibody – doi: 10.1038/s41467-023-38943-2

Eukaryotic cell lines

Policy information about cell lines and Sex and Gender in Research

Cell line source(s)	BS-C-1 cells (ATCC #CCL-26; from African green monkey's kidney; sex unknown or irrelevant)
Authentication	The cell line used was not authenticated.
Mycoplasma contamination	The cell line used was not tested for mycoplasma contamination.
Commonly misidentified lines (See ICLAC register)	No commonly misidentified cell lines were used.

Plants

Seed stocks	No plant specimens were used.
Novel plant genotypes	See above.
Authentication	See above.
